# A Case Report on the Successful Treatment of* Streptococcus pneumoniae*-Induced Infectious Abdominal Aortic Aneurysm Initially Presenting with Meningitis

**DOI:** 10.1155/2015/825069

**Published:** 2015-12-08

**Authors:** Yohei Kawatani, Yoshitsugu Nakamura, Yujiro Hayashi, Tetsuyoshi Taneichi, Yujiro Ito, Hirotsugu Kurobe, Yuji Suda, Takaki Hori

**Affiliations:** Department of Cardiovascular Surgery, Chiba-Nishi General Hospital, 107-1 Kanegasaku, Matsudo-Shi, Chiba-Ken 2702251, Japan

## Abstract

Infectious abdominal aortic aneurysms often present with abdominal and lower back pain, but prolonged fever may be the only symptom. Infectious abdominal aortic aneurysms initially presenting with meningitis are extremely rare; there are no reports of their successful treatment. Cases with* Streptococcus pneumoniae* as the causative bacteria are even rarer with a higher mortality rate than those caused by other bacteria. We present the case of a 65-year-old man with lower limb weakness and back pain. Examination revealed fever and neck stiffness. Cerebrospinal fluid showed leukocytosis and low glucose levels. The patient was diagnosed with meningitis and bacteremia caused by* Streptococcus pneumoniae* and treated with antibiotics. Fever, inflammatory response, and neurologic findings showed improvement. However, abdominal computed tomography revealed an aneurysm not present on admission. Antibiotics were continued, and a rifampicin soaked artificial vascular graft was implanted. Tissue cultures showed no bacteria, and histological findings indicated inflammation with high leukocyte levels. There were no postoperative complications or neurologic abnormalities. Physical examination, blood tests, and computed tomography confirmed there was no relapse over the following 13 months. This is the first reported case of survival of a patient with an infectious abdominal aortic aneurysm initially presenting with meningitis caused by* Streptococcus pneumoniae*.

## 1. Introduction

Infectious abdominal aortic aneurysm was reported in 1851 by Osler as a rare and lethal disease [1]. Infectious aneurysms comprise 1% to 3% of all aneurysms [2]. Eighteen percent of infectious aortic aneurysms are thought to be abdominal [3].

Abdominal and lower back pain are the most common initial symptoms of infectious abdominal aortic aneurysms, but in some cases, fever is the only symptom at onset. Infectious aneurysms with neurologic symptoms or meningitis as the initial symptoms are extremely rare, with very few case reports in the literature. In addition, there are no case reports in the literature to date documenting successful treatment of such a case.

*Streptococcus pneumoniae (S. pneumoniae)* and* Neisseria meningitidis* are the causative bacteria in over 80% of community-acquired meningitis cases [4], but the typical causative bacteria for infectious abdominal aortic aneurysms are* Salmonella* and* Staphylococcus* [2], with* S. pneumoniae* being rare for this condition.

A 65-year-old man was diagnosed with community-acquired meningitis caused by* S. pneumoniae* and received treatment; during his clinical course, he also received a new diagnosis of an infectious abdominal aortic aneurysm, for which surgery was performed. The patient was successfully treated at our institution without any residual neurologic or cardiovascular symptoms, and this report documents the case.

## 2. Case Presentation

**Figure 1:**
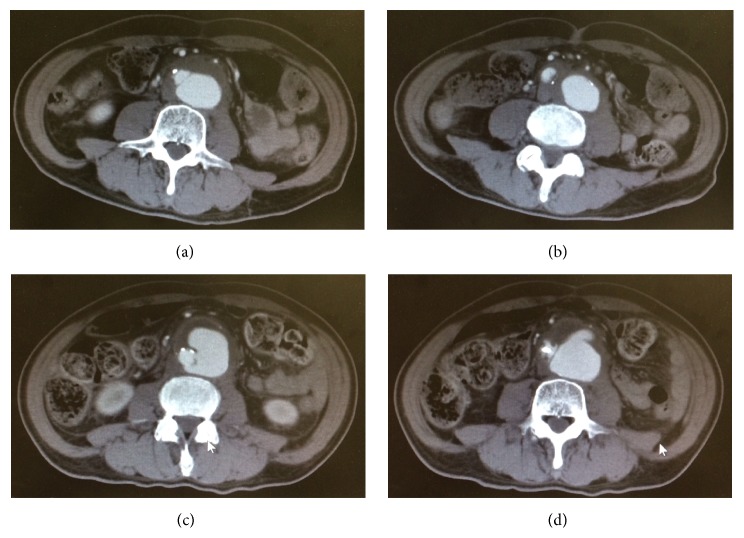
(a) and (b) Computed tomography scans taken during the patient's initial examination. (c) and (d) Computed tomography scans taken on day 9 of the illness. We observed enlargement of the abdominal aortic aneurysm and thickening of the arterial wall.

A 65-year-old man presented to the emergency room complaining of back pain, left lower limb weakness, altered mental condition, and neck stiffness. Vital signs were stable other than a fever. Blood test results revealed a leukocyte count of 23600/mm^3^ and a serum C-reactive protein (CRP) level of 12.7 mg/dL. A cerebrospinal fluid (CSF) examination revealed leukocytosis and a low glucose level (white cell count was 1616 cells/mm^3^, glucose was 6 mmol/L, and protein rate was 563 g/L). CSF and blood culture were positive for* S. pneumoniae*. A computed tomography (CT) scan showed a 45 × 49 mm abdominal aortic aneurysm. Meningitis with* S. pneumoniae *as the causative bacteria was diagnosed. The patient was treated with meropenem, vancomycin, and ampicillin. On hospital day 14, when we received the sensitivity results, we stopped the vancomycin and continued meropenem. We continued meropenem preoperatively and postoperatively. The patient stabilized on postoperative day 21, and we changed meropenem to ampicillin and sulbactam, which was administered for 3 weeks. After that, we prescribed levofloxacin. After the initiation of treatment, the patient's neurological findings gradually improved. However, the patient experienced anorexia during the treatment. A follow-up whole body CT scan was performed on hospital day 9 to evaluate the abdominal symptoms, which showed rapid growth of the aneurysm to 56 × 66 mm (Figure 1). The aneurysm had become saccular with inflammatory changes such as increased density of the surrounding fat, and the mantle sign was positive.

We diagnosed the patient with a mycotic aneurysm. We performed an urgent operation to repair the abdominal aortic aneurysm. An artificial vascular graft was soaked for 20 minutes in 0.1% rifampicin. During the operation, the patient remained hemodynamically stable. Intraoperative findings showed an enlarged aorta with adhesions to the surrounding tissue. There was an abscess in the aneurysmal wall. The abscess and necrotic tissue around the aneurysm were debrided, and reconstruction was performed with a rifampicin soaked bifurcated J graft. We also performed omentoplasty. The graft was wrapped with omentum to the left gastroepiploic artery pedicle.

Tissue cultures did not reveal any bacterial organisms, possibly because antibiotic therapy had already been administered. Pathologic examination of the aorta revealed leukocytosis.

The patient was transferred to the intensive care unit postoperatively, and his course was uneventful. The patient underwent extubation 15 hours after surgery and was given oral intake 5 days after surgery.

After the operation, the inflammatory findings improved. The serum CRP levels were 16.39, 30.98, 21.67, and 4.87 mg/dL, and the white blood cell (WBC) counts were 16900, 17440, 12490, and 10380/*μ*L on postoperative days 1, 2, 3, and 7, respectively. The body temperature returned to normal on postoperative day 3. He was discharged without any neurological, gastrointestinal, or renal dysfunctions or any other organ disorder.

**Figure 2:**
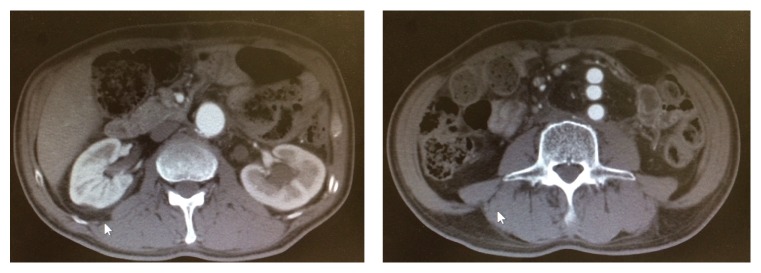
Computed tomography scans taken when the patient was discharged. No leakage of blood into the area around the artificial vascular graft or residual abscess was seen. The omentum with intact blood flow can be seen in the area around the graft.

At a follow-up visit 13 months after surgery, he presented without any complaints. We did not detect any neurological dysfunction, and a blood test revealed a CRP level of 0.07 mg/dL and WBC count of 5350/*μ*L (Figure 2).

## 3. Discussion

Infectious abdominal aortic aneurysms are a rare condition and comprise 0.5–1.3% of aneurysms for which surgery is performed [6]. The condition causes various symptoms, with classical symptoms including fever, abdominal pain, back pain, testicular pain, and a pulsatile abdominal mass. However, when only nonspecific symptoms are present, diagnosis of the condition is difficult and incurs delays [7]. The symptoms of infectious abdominal aortic aneurysm caused by* S. pneumoniae* do not differ significantly from those caused by other bacteria, with patients commonly complaining of fever, back pain, hypophagia, and other symptoms [8]. Cases of infectious abdominal aortic aneurysm like ours, in which the patient presented initially with community-acquired bacterial meningitis and bacteremia, are extremely rare, with no reports discussing their frequency. It is possible that diagnosis of these infectious abdominal aortic aneurysms is delayed, or in some cases, they are not diagnosed at all due to a separate diagnosis and treatment of meningitis.

*S. pneumoniae* and* Neisseria meningitidis* are the causative bacteria for a large proportion of community-acquired meningitis—50% and 25% of cases, respectively. The mortality rate from* S. pneumoniae*-induced bacterial meningitis is high at 19–37% [4]. Gram-positive bacteria are common as the causative bacteria for infectious abdominal aortic aneurysms, comprising 60% of cases. Of these,* Staphylococcus aureus* and* Salmonella* species comprise a large proportion at 46% and 8%, respectively [2]. Development of an infection in an existing atherosclerotic aneurysm due to bacteremia is thought to be the principal infection route for these bacteria [9].

It is thought that* S. pneumoniae* was often the causative bacteria for infectious endocarditis and other infectious diseases of the cardiovascular system before antibiotics became widely available [10]. However, due to its high sensitivity to penicillin,* S. pneumoniae* has been extremely rare as the causative bacteria for cardiovascular infections since the use of antibiotics has become widespread. This is thought to be one of the reasons for the rarity of* S. pneumoniae*-induced infectious abdominal aortic aneurysms. According to a report by Cartery et al., a review of the English-language literature up to 2011 revealed that infectious abdominal aortic aneurysms caused by* S. pneumoniae *had only been reported in 30 cases (Table 1) [5]. Searching on Medline for the keywords* meningitis*,* infectious aneurysm*,* mycotic aneurysm*, and* aortic aneurysm* revealed three case reports relating to infectious abdominal aortic aneurysms that initially presented with meningitis. The addition of our case brings the total number of reports to four. While* S. pneumoniae* as the causative bacteria for infectious abdominal aortic aneurysms is rare, it was the causative bacteria in three of these four cases (Table 2). Although the small sample size precludes statistical conclusions from being drawn, there may be some correlation between* S. pneumoniae* and infectious abdominal aortic aneurysms with meningitis as the initial symptom. Further investigation is required on this matter.

*S. pneumoniae* is commonly treated with broad-spectrum penicillins or cephalosporins. However, in recent years, the emergence of* S. pneumoniae* that is resistant to treatment with antibiotics has been reported [11]. In this case, meropenem and vancomycin were administered to cover for any potential resistant bacteria until we had identified the bacteria by the blood and cerebrospinal fluid cultures, which were performed when the patient was admitted to the hospital and the results of the sensitivity test were revealed. Once* S. pneumoniae* had been detected, this regimen was replaced with a meropenem-only treatment regimen. The same drug was continued until the postoperative period, at which time meropenem was tapered and ampicillin/sulbactam was administered as indicated by the sensitivity test results. Administration of this combination was continued until the sixth postoperative week, even after blood test findings had improved.

When bacterial meningitis is suspected, swift diagnosis and commencement of antibiotics are recommended [12]. Furthermore, early administration of antibiotics ultimately acted as the preoperative antibiotics for the infectious abdominal aortic aneurysm. While a reaction suggesting inflammation was observed from the abdominal aortic aneurysm tissue, the culture was negative. A negative tissue culture due to the administration of preoperative antibiotics is commonly seen.

The survival rate for infectious abdominal aortic aneurysms differs significantly depending on the treatment method. While a literary review of infectious abdominal aortic aneurysm with* S. pneumoniae *as the causative bacteria indicated a survival rate of 84%, cases treated with only antibiotics or only surgery or with no treatment had a survival rate of 0% [5]. Both administration of antibiotics and surgery are necessary for treatment of this condition.

Generally, the surgical method used to treat infectious abdominal aortic aneurysms is repair by open surgery. Soaking the graft in rifampicin is thought to lower the risk of postoperative infection [13], and we believe this technique should be used wherever possible. Wrapping the graft with omentum has been reported to reduce the incidence of postoperative graft infections [14].

In recent years, treatment with an endograft has been reported [15]. However, there are also reports of endograft infections bringing about tragic results. For veins infected with* S. aureus*, an animal experimental model comparing conventional techniques to polytetrafluoroethylene grafts using the endovascular technique indicated that endograft recipients were more vulnerable to infection than those in whom the conventional technique had been used. The authors stated that this is because resistance to infection is greater in the extravascular retroperitoneum than the arterial wall [16]. Furthermore, we believe that open surgery also allows for better debridement of infected or necrotic tissue than endograft surgery.

Postoperative graft infection is a lethal complication that is often extremely difficult to treat. Considering the gravity of an event such as graft infection, we believe that from the first surgery a complete cure should be prioritized over minimal invasiveness, and thus we do not recommend the endograft procedure at this time.

Delaying surgery can lead to unnecessary loss of life. However, in some cases, early surgery may not be possible, as implanting an artificial vascular graft at the peak of meningitis or bacteremia infection may increase the risk of infection relapse. In these cases, performing emergency endovascular aneurysm repair (EVAR) to stabilize the hemodynamics and then subsequently performing debridement and implanting the artificial vascular graft at a later point could improve the survival rate. Depending on the circumstances, EVAR may be an effective bridge to open surgery in cases of infectious abdominal aortic aneurysm.

It is considered beneficial for postoperative antibiotics to target bacteria detected from samples taken pre- and intraoperatively, but as these drugs are administered over a long period of time, the development of a new drug resistance is also possible. When exacerbation of infection is observed postoperatively, it is necessary to repeat blood cultures to confirm whether microbial substitution has taken place and consider changing the antibiotic in light of sensitivity tests.

There is no consensus regarding the time period for postoperative administration of antibiotics. In most cases, antibiotics are generally administered for 6–12 weeks. At this institution, drugs are administered for 6 weeks or more, with negative blood cultures and normalization of serum CRP levels and leukocytes used as a guide for the cessation of treatment.

Treatment for* S. pneumoniae*-induced infectious abdominal aortic aneurysm with meningitis as its initial symptom must center on appropriate administration of antibiotics to treat the meningitis and a simultaneous investigation of surgical treatment methods as soon as the infectious abdominal aortic aneurysm is observed. In addition, surgery must be performed at an appropriate time before hemodynamic collapse.

Using Medline to search for the keywords* aortic aneurysm, mycotic aneurysm, infectious aneurysm*, and* meningitis* produced reports for only three cases of infectious abdominal aortic aneurysm with onset from meningitis [5, 17]. Furthermore, all patients had died during the treatment course, which means there was no reported instance in the literature of treatment achieving survival (Table 2). This is the only report in the English literature to document a case of successful treatment of infectious abdominal aortic aneurysm, and it is also significant because the patient has experienced no relapse on a follow-up visit more than 1 year later.

## Figures and Tables

**Table 1:** Reported cases of infectious abdominal aortic aneurysm caused by *Streptococcus pneumoniae*.

Case	Age, gender	Symptoms	Positive imaging study	Blood culture	Source of infection	Treatment	Outcome
(1) 1945	60, M	Fever	?	−	Pneumonia	Antibiotics	Died

(2) 1966	76, F	Fever, back pain	?	+	Unknown	Antibiotics	Died

(3) 1983	48, M	Fever, back pain	CT bone scan	+	Pneumonia	Antibiotics, surgery	Survived

(4) 1985	48, M	Fever, abdominal pain	CT	+	Unknown	Antibiotics, surgery	Survived

(5) 1988	51, M	Fever, back and abdominal pain	?	+	Unknown	None	Died

(6) 1988	71, F	Fever, abdominal pain	CT gallium	−	Endocarditis, septic joint	Antibiotics, surgery	Died

(7) 1991	87, M	Back pain	CT angiogram	−	Unknown	Antibiotics, surgery	Survived

(8) 1992	59, F	Fever, back pain	CT	+	Unknown	Antibiotics, surgery	Survived

(9) 1992	77, M	Back pain	CT	−	Unknown	Antibiotics, surgery	Survived

(10) 1993	?	Fever	?	+	Unknown	Antibiotics, surgery	Survived

(11) 1995	67, M	Unknown	CT	+	Unknown	Antibiotics, surgery	Survived

(12) 1997	30, M	Fever, cough	CT angiogram	+	Pneumonia	Antibiotics, surgery	Survived

(13) 1997	62, M	Fever, back pain, and groin pain	Autopsy	+	Pneumonia, meningitis	Antibiotics	Died

(14) 1998	54, M	Fever	CT MRI	+	Unknown	Antibiotics	Survived

(15) 1998	62, F	Fever, back pain	?	− (tissue culture +)	Epidural abscess	Antibiotics	Survived

(16) 1998	54, M	Fever, back pain	?	− (tissue culture +)	Endocarditis, meningitis	Antibiotics	Died

(17) 1999	60, M	Fever, back pain	CT MRI	+	Unknown	Antibiotics, surgery	Died

(18) 1999	52, F	Fever, back pain	CT	+	Spondylodiscitis	Antibiotics	Died

(19) 1999	65, F	Abdominal and back pain, weight loss	CT abdominal ultrasonography	− (tissue culture +)	Pneumonia	Antibiotics, surgery	Survived

(20) 2001	60, F	Asymptomatic	Ultrasonography	− (tissue culture +)	Unknown	Antibiotics, surgery	Survived

(21) 2001	66, M	Abdominal pain, fever	Ultrasonography	− (tissue culture +)	Unknown	Antibiotics, surgery	Survived

(22) 2001	69, F	Fever, back pain	CT	− (tissue culture +)	Unknown	Antibiotics, surgery	Survived

(23) 2002	30, F	Fever, back pain	CT	+ (tissue culture +)	Unknown	Antibiotics, surgery	Survived

(24) 2003	72, M	Fever, conscious disturbance	CT angiogram	+	Unknown	Antibiotics, surgery	Survived

(25) 2004	69, F	Fever, shoulder pain, abdominal pain, and diarrhea	CT	+	Unknown	Antibiotics, surgery	Survived

(26) 2004	62, M	Fever, cough, and testicular abdominal pain	CT MRI	+	Unknown	Antibiotics, surgery	Survived

(27) 2005	44, M	Back and groin pain	Angiogram	+	Unknown	Antibiotics, surgery	Survived

(28) 2006	72, M	Abdominal and back pain, fever	CT	− (tissue culture +)	Unknown	Antibiotics, surgery	Survived

(29) 2006	69, F	Back pain	CT	− (PCR +)	Unknown	Antibiotics, surgery	Survived

(30) 2007	75, M	Fever abdominal pain, anorexia, and weight loss	CT	+	Pneumonia	Antibiotics, surgery	Died

Our case	63, M	Weakness, back pain, and consciousness disorder	CT	+	Meningitis	Antibiotics, Surgery	Survived

Cited from [5] with our case added.

*S. pneumoniae: Streptococcus pneumoniae*.

**Table 2:** Reported cases of infectious abdominal aortic aneurysm with onset from meningitis.

Case	Age, gender	Symptoms	Positive imaging study	Blood culture	Causative organism	Treatment	Outcome	Cause of death
(1) 1997	62, M	Fever, headache, cough, and nausea	None (autopsy)	+	*S. pneumonia*	Antibiotics	Died	Hemorrhage

(2) 2008	65, F	Weakness, fever, and neck stiffness	CT	+	*S. pneumonia*	Antibiotics, surgery	Died	Hemorrhage

(3) 2011	59, M	Consciousness disorder, fever	CT	+	*E. coli*	Antibiotics, surgery	Died	Hemorrhage

Our case	63, M	Weakness, back pain, and consciousness disorder	CT	+	*S. pneumonia*	Antibiotics, surgery	Survived	—

*S. pneumoniae*: *Streptococcus pneumonia*.

*E. coli*: *Escherichia coli*.
